# Supplementary material for: Single-particle imaging of stress-promoters induction reveals the interplay between MAPK signaling, chromatin and transcription factors
Source: Nat Commun. 2020 Jun 23;11:3171. doi: 10.1038/s41467-020-16943-w (PMC7311541; doi:10.1038/s41467-020-16943-w)
Supplement: Supplementary file 1 — Supplementary Information [file 41467_2020_16943_MOESM1_ESM.pdf]

Supplementary Materials to:

Single-particle imaging of stress-promoters induction reveals the interplay between MAPK signaling, chromatin and transcription factors

Wosika et al.
Department of Fundamental Microbiology,
University of Lausanne

Supplementary Tables

Supplementary Table 1. Plasmids used in this study

Plasmid name	Description	Backbone
pSP264	pSTL1 24xPP7sl	pHIS [a]
pSP266	pCTT1 24xPP7sl	pHIS [a]
pVW200	pGPD1 24xPP7sl	pHIS [a]
pVW293	pHSP12 24xPP7sl	pHIS [a]
pVW294	pGRE2 24xPP7sl	pHIS [a]
pVW295	pALD3 24xPP7sl	pHIS [a]
pSP568	pSTL1 24xMS2sl	pHIS [a]
pVW284	pADH1 PP7ΔFG-GFPenvy tCYC1	pSIV URA3 [b]
pVW300	pTEF PP7ΔFG-GFPenvy tCYC1	pSIV URA3 [b]
pVW296	pADH1 PP7ΔFG-mCherry tCYC1	pSIV URA3 [b]
pSP561	pADH1 MS2-GFPenvy tCYC1	pSIV URA3 [b]
pSP571	pGPD Cas9 / sgRNA ($STL1_{62}$)	pRS423II [c]
pSP569	pSTL1 24xPP7sl $STL1_{100-600}$	pHIS [d]
pSP566	mCherry-CaaX	pGT TRP1 [e]

[a] Modified from Larson *et al.*[1] Integrates in the *GLT1* locus.
[b] pSIV vector from Wosika *et al.*[2].
[c] Modified from Laughery *et al.*[3]
[d] Used as repair DNA for CRISPR transformation.
[e] Modified from Wosika *et al.*[2].

Supplementary Table 2. List of yeast strains

All strains were constructed in the W303 background (ySP2) *MAT*a *leu2-3,112 trp1-1 can1-100 ura3-1 ade2-1 his3-11,15*. [4]

Strain name	Relevant Genotype	Ancestor strain
ySP269	HTA2-mCherry:URA3	ySP2
ySP329	HTA2-mCherry:URA3 Hog1-GFP:HIS3	ySP269
yED215	HTA2-mCherry:URA3	ySP2
yVW401	HTA2-mCherry:URA3<br>pSIVu pADH1 PP7ΔFG-GFPenvy tCYC1::URA3	yED215
yVW403	HTA2-mCherry:URA3<br>pSIVu pADH1 PP7ΔFG-GFPenvy tCYC1::URA3<br>pSTL1 24xPP7sl GLT1 tGLT1::GLT1:HIS3	yVW401
yVW428	HTA2-mCherry:URA3<br>pSIVu pADH1 PP7ΔFG-GFPenvy tCYC1::URA3<br>pCTT1 24xPP7sl GLT1 tGLT1::GLT1:HIS3	yVW401
yVW429	HTA2-mCherry:URA3<br>pSIVu pADH1 PP7ΔFG-GFPenvy tCYC1::URA3<br>pHSP12 24xPP7sl GLT1 tGLT1::GLT1:HIS3	yVW401
yVW430	HTA2-mCherry:URA3<br>pSIVu pADH1 PP7ΔFG-GFPenvy tCYC1::URA3<br>pGRE2 24xPP7sl GLT1 tGLT1::GLT1:HIS3	yVW401
yVW431	HTA2-mCherry:URA3<br>pSIVu pADH1 PP7ΔFG-GFPenvy tCYC1::URA3<br>pALD3 24xPP7sl GLT1 tGLT1::GLT1:HIS3	yVW401
yVW432	HTA2-mCherry:URA3<br>pSIVu pADH1 PP7ΔFG-GFPenvy tCYC1::URA3<br>pGPD1 24xPP7sl GLT1 tGLT1::GLT1:HIS3	yVW401
yVW409	HTA2-mCherry:URA3<br>pSIVu pADH1 PP7ΔFG-GFPenvy tCYC1::URA3<br>pSTL1 24xPP7sl GLT1 tGLT1::GLT1:HIS3<br>GCN5::NAT	yVW403
yVW416	HTA2-mCherry:URA3<br>pSIVu pADH1 PP7ΔFG-GFPenvy tCYC1::URA3<br>pSTL1 24xPP7sl GLT1 tGLT1::GLT1:HIS3<br>HTZ1::NAT	yVW403
yVW405	HTA2-mCherry:URA3<br>pSIVu pADH1 PP7ΔFG-GFPenvy tCYC1::URA3<br>pSTL1 24xPP7sl GLT1 tGLT1::GLT1:HIS3<br>HOT1::NAT	yVW403
yVW407	HTA2-mCherry:URA3<br>pSIVu pADH1 PP7ΔFG-GFPenvy tCYC1::URA3<br>pSTL1 24xPP7sl GLT1 tGLT1::GLT1:HIS3<br>SKO1::NAT	yVW403
ySP915	HTA2-mCherry:URA3<br>pSIVu pADH1 PP7ΔFG-GFPenvy tCYC1::URA3<br>pSTL1 24xPP7sl GLT1 tGLT1::GLT1:HIS3<br>MSN4::KAN, MSN4::NAT	yVW403
yVW471	HTA2-mCherry:URA3<br>pSIVu pADH1 PP7ΔFG-GFPenvy tCYC1::URA3<br>pGPD1 24xPP7sl GLT1 tGLT1::GLT1:HIS3<br>HOT1::NAT	yVW432
yVW472	HTA2-mCherry:URA3<br>pSIVu pADH1 PP7ΔFG-GFPenvy tCYC1::URA3<br>pGPD1 24xPP7sl GLT1 tGLT1::GLT1:HIS3<br>SKO1::NAT	yVW432
ySP918	HTA2-mCherry:URA3<br>pSIVu pADH1 PP7ΔFG-GFPenvy tCYC1::URA3<br>pGPD1 24xPP7sl GLT1 tGLT1::GLT1:HIS3<br>MSN4::KAN, MSN4::NAT	yVW432

yVW476	HTA2-mCherry:URA3<br>pSIVu pTEF PP7ΔFG-GFPenvy tCYC1::URA3<br>pGPD1 24xPP7sl GLT1 tGLT1::GLT1:HIS3	yVW454
yVW477	HTA2-mCherry:URA3<br>pSIVu pTEF PP7ΔFG-GFPenvy tCYC1::URA3<br>pHSP12 24xPP7sl GLT1 tGLT1::GLT1:HIS3	yVW454
yVW474	HTA2-tdiRFP:ADE<br>pSIVu pADH1 PP7ΔFG-GFPenvy tCYC1::URA3<br>pSTL1 24xPP7sl GLT1 tGLT1::GLT1:HIS3<br>HOG1-mCherry: LEU2	
ySP919	HTA2-tdiRFP:ADE<br>pSIVu pADH1 PP7ΔFG-GFPenvy tCYC1::URA3<br>pSTL1 24xPP7sl GLT1 tGLT1::GLT1:HIS3<br>HOG1-mCherry-CaaX: TRP1	
ySP921	HTA2-tdiRFP:NAT<br>pSIVu pADH1 PP7ΔFG-GFPenvy tCYC1::URA3<br>pGPD1 24xPP7sl GLT1 tGLT1::GLT1:HIS3<br>HOG1-mCherry: LEU2	
ySP922	HTA2-tdiRFP:NAT<br>pSIVu pADH1 PP7ΔFG-GFPenvy tCYC1::URA3<br>pGPD1 24xPP7sl GLT1 tGLT1::GLT1:HIS3<br>HOG1-mCherry-CaaX: TRP1	
ySP929	HTA2-mCherry:URA3<br>pSIVu pADH1 PP7ΔFG-GFPenvy tCYC1::URA3<br>pSTL1 24xPP7sl :STL1 (clone 8)	yVW401
ySP930	HTA2-mCherry:URA3<br>pSIVu pADH1 PP7ΔFG-GFPenvy tCYC1::URA3<br>pSTL1 24xPP7sl :STL1 (clone 10)	yVW401
ySP927	MAT**a** / MATα<br>Hta2-tdiRFP:NAT / Hta2-tdiRFP :TRP<br>pSTL1 24xPP7sl GLT1 tGLT1::GLT1:HIS3 /<br>pSTL1 24xMS2sl GLT1 tGLT1::GLT1:HIS3<br>pSIVu pADH1 PP7ΔFG-GFPenvy tCYC1::URA3 /<br>pSIVu pADH1 MS2-GFPenvy tCYC1::URA3	
ySP884	HTA2-CFP:HIS3<br>HOG1-mCitrine:LEU2<br>MSN2-mCherry:URA3	
ySP763	HTA2-CFP:HIS3<br>HOG1-mCitrine:LEU2<br>pSIVu pSTL1-dPSTR-mCherry::URA3	
ySP764	HTA2-CFP:HIS3<br>HOG1-mCitrine:LEU2<br>pSIVu pHSP12-dPSTR-mCherry::URA3	
yVW418	HTA2-CFP:HIS3<br>HOG1-mCitrine:LEU2<br>pSIVu pALD3-dPSTR-mCherry::URA3	
ySP766	HTA2-CFP:HIS3<br>HOG1-mCitrine:LEU2<br>pSIVu pCTT1-dPSTR-mCherry::URA3	

Supplementary Table 3. List of primers

	Oligo	GENE	Orientation	Sequence	Information
Promoter	**oFR3808**	STL1	forward	GTATCGgagctcGCAGAACACCACTACTGATACTC	Promoter -800 bp
	**oFR3809**	STL1	reverse	GTATCGtctagaGGTCTAAAACTTTCTATGTTCTATTTTTC	Promoter 0kB
	**oFR4372**	CTT1	forward	GTATCGGAGCTCTTGCCAAGTACATAGAATCC	Promoter -1kB
	**oFR4373**	CTT1	reverse	GTATCGACTAGTTTGTGAAGCTGAGCTGATTG	Promoter 0kB
	**oFR4370**	GRE2	forward	GTATCGGAGCTCAATATGTAATAGTTATGCAC	Promoter -1kB
	**oFR4371**	GRE2	reverse	GTATCGACTAGTATTTACGGGCGTGTGATACT	Promoter 0kB
	**oFR3804**	ALD3	forward	GTATCGgagctcTTATGTAAGTGCGTAAGTACCTAAATAG	Promoter -664 bp
	**oFR3805**	ALD3	reverse	GTATCGtctagaTTTTCTTTTGGCTAATTTTCTAAATG	Promoter 0kB
	**oVW1540**	HSP12	forward	GTATCGGAGCTCTGATATAGGACTTCTCTCTCTTTT	amplify pHSP12 (full length=1kb) from gDNA
	**oSP1546**	HSP12	reverse	CGATACCCGCGGTGTTGTATTTAGTTTTTTTTGTTT	To amplify pHSP12 and clone SacI SacII
	**oDA501**	GPD1	forward	GTATCGGAGCTCGAAGCCCGAAAGAGTTATCG	For Amplification of pGPD1 between SacI and XbaI.
	**oDA502**	GPD1	forward	CGATACTCTAGACTTTATATTATCAATATTTGTGTTTGTG	For Amplification of pGPD1 between SacI and XbaI.
	**oSP061**	GLT1	reverse	CTCCGGTTGTTTTTCATC	In gene 600 To verify pp7 SL integration
	**oDA160**	STL1	forward	GAAATTGAGAAAGCTTAAGT	For sequencing 200nt before the end of pSTL1
	**oVW447**	GLT1	reverse	CTCGAGATTCACTAAAAGATATCTAGCGTCAGTAACAAT	Reverse primer to amplify 250bp from GLT1 start
Deletion	**oDA810**	HOT1	forward	AAAAGATTATATTTAGGGTACATATGGCTGGAGCATAATGCGTACGCTGCAGGTCGAC	Deletion NAT
	**oDA811**	HOT1	reverse	CCTATGATTGTAAACGATTATTTACTATCGTACGTGCCTAATCGATGAATTCGAGCTCG	Deletion NAT
	**oDA816**	HOT1	forward	AACCGAGAAGCAGCGATTCT	Primer -500 into promoter to check deletion
	**oDA817**	HOT1	reverse	AAAGCCCTACTCTACAAAAGAAAA	Primer +500 in terminator to check deletion
	**oSP390**	TEF	reverse	GTATTCTGGGCCTCCATGTC	TEF promoter Verify NAT/KAN cassette integration
	**oSP062**	TEF	forward	TGGTCGCTATACTGCTGTCG	TEF terminator Verify NAT/KAN cassette integration
	**oSP402**	HOG1	forward	AAAGGGAAAACAGGGAAAACTACAACTATCGTATATAATAATGCGTACGCTGCAGGTCGAC	NAT /Kan deletion Hog1
	**oSP403**	HOG1	reverse	GAAGTAAGAATGAGTGGTTAGGGACATTAAAAAAACACGTTTAATCGATGAATTCGAGCTCG	NAT /Kan deletion Hog1
	**oDA224**	GCN5	forward	AAGTCTTCAGTTAACTCAGGTTCGTATTCTACATTAGATGCGTACGCTGCAGGTCGAC	Deletion NAT
	**oDA225**	GCN5	reverse	TCGAAAGGAATAGTAGCGGAAAAGCTTCTTCTACGCATTAATCGATGAATTCGAGCTCG	Deletion NAT
	**oDA1095**	GCN5	forward	TCAACGAATGAATACGTACA	check deletion of gcn5. -500 of GCN5 ORF.
	**oDA484**	GCN5	reverse	AGACGGATTCAAAATATATGATAACGA	+250 after stop of GCN5 to check deletion.
	**oVW1250**	HTZ1	forward	AATTTCGCACTATAGCCGCACGTAAAAATAACTTAACATAAGCGGATGCCGGGAGCAGAC	To make a HTZ1 delta strain.
	**oVW1251**	HTZ1	reverse	AGGGAGAATTACGGGAAATGGGAAAGAAAAACTATTCTTCGTGAGCTGATACCGCTCGCC	To make a HTZ1 delta strain.
	**oVW1256**	HTZ1	forward	TGATTAATTAACCTCGGAGGTGTTACC	To check the deletion -500 to ATG. Tm 57.
	**oDA814**	SKO1	forward	ATACACCTGCCCAGTCTCTAGACCCTGCTTAATCATTATGCGTACGCTGCAGGTCGAC	Deletion NAT
	**oDA815**	SKO1	reverse	AGATAGAAGACTATTTAAGAACCCCGTCGCTATCTCGTCAATCGATGAATTCGAGCTCG	Deletion NAT
	**oDA821**	SKO1	reverse	CCAAGTCGTACTTAACCGC	Primer +500 in promoter to check deletion
	**oDA820**	SKO1	forward	CCATCTCTTGAAAGCGAACAAA	Primer -500 into terminator to check deletion
	**oSP1691**	MSN2	forward	GCGGAAAAGAAAAACAAGACACCG	500 before start
	**oSP1692**	MSN2	reverse	GAGGCCTAGGAAATTAAGTATTCGGC	500 After Stop
	**oSP1705**	MSN2	forward	CGTTCCTGGCCAGACGGC	750 before START
	**oSP1706**	MSN2	reverse	GGGCTTTACCGCTAATAAGTGGAGTA	650 After STOP
	**oSP1693**	MSN4	forward	GGCTTTCTCCCACGAGGTTT	500 before start
	**oSP1694**	MSN4	reverse	GCCTTTTTCCCTACTCCTTTTAGTACG	500 After Stop
	**oSP1711**	MSN4	forward	GGCATTTTGAGCGCGGCAAAA	750 before START
	**oSP1712**	MSN4	reverse	CCAACCAAGCCTCATTGCTCCTT	800 After STOP

Tagging	**oSP645**	HTA2	forward	ACTTGTTGCCAAAGAAGTCTGCCAAGACTGCCAAAGCTTCTCAAGAACTGGCGGCCGCTCTAGAACTA	pGT tagging of Hta2
	**oED830**	HTA2	reverse	CGTAACAAAAGAAAGAGAGCCTAGCTGTAATATATCTTTATAACATGTATATGGAAAAACGCCAGCAACG	Reverse primer to tag Hta2.
	**oVW853**	HOG1	forward	CGGTAACCAGGCCATACAGTACGCTAATGAGTTCCAACAGGCGGCCGCTCTAGAACTA	To tag hog1 with the pGT plasmids.
	**oVW862**	HOG1	reverse	GCTGATAAACAAACAATACGCCATAAGTGACGGTTCTTGGATGGAAAAACGCCAGCAACG	To tag hog1 with the pGT plasmids.
	**oSP1642**	MSN2	forward	GCGATAATTTGTCGCAACACATCAAGACTCATAAAAAACATGGAGACATTGCGGCCGCTCTAGAACTA	pGT tagging
	**oSP1643**	MSN2	reverse	GCGGGGGAACTGAATTCCTTCAAAATGTGAAGGTACCGGAAAAATGGCAAATGGAAAAACGCCAGCAACG	pGT tagging
	**oSP1648**	RAS2	forward	CTAGCAGGATCGGGTGGCTGTTGTATTATAAGTTAAG	To clone Ras2 CAAX motif into pGTL-plasmid
	**oSP1649**	RAS2	reverse	TCGACTTAACTTATAATACAACAGCCACCCGATCCTG	To clone Ras2 CAAX motif into pGTL-plasmid
STL1 endogenous	**oSP1697**	STL1	forward	GTATCGAGATCTGATATCCGCATCTATGACGGGCTTCTC	+100bp after STL1 ATG to clone STL1 after PP7sl
	**oSP1698**	STL1	reverse	GTATCGGCGGCCGCCTCCACTGAACAGAACTGTTGGTATAAG	+600bp after STL1 ATG to clone STL1 after PP7sl
	**oSP1699**	STL1	forward	ATAAGCAGAACCAGTCACTGGTTTTAGAG	sgOligo to target STL1 locus in gene at 62bp after start to integrate PP7 loops
	**oSP1700**	STL1	reverse	CTAGCTCTAAAACCAGTGACTGGTTCTGCTTATACGT	sgOligo to target STL1 locus in gene at 62bp after start to integrate PP7 loops
	**oSP1701**	STL1	forward	CACACTCATAGTATATAAACAAGCCC	Primer 100 before ATG. To verify integration of PP7 loops
	**oSP1702**	STL1	reverse	AACGATTTGCATTGACACGG	Primer 650 after ATG. To verify integration of PP7 loops

Supplementary Table 4. Summary of source data, strains and cell numbers

	Strain	Stress	Replicates	Date yymmdd	Exp Num	Nb Cells
**Figure 1 d**	yVW403	0.0 M NaCl	rep1	181120	3739	313
	yVW403	0.1M NaCl	rep1	181120	3745	404
	yVW403	0.2M NaCl	rep2	181127	3762	229
	yVW403	0.3M NaCl	rep3	190124	3849	201
**Figure 2 a**	yVW431	0.2M NaCl	rep1	181127	3752	171
	yVW428	0.2M NaCl	rep3	190118	3827	140
	yVW403	0.2M NaCl	rep2	181127	3762	229
	yVW430	0.2M NaCl	rep1	181127	3754	289
	yVW429	0.2M NaCl	rep4	190329	3951	243
	yVW432	0.2M NaCl	rep2	181127	3758	335
**Figure 2 f**	yVW403	0.2M NaCl	rep2	181127	3762	229
	yVW409	0.2M NaCl	rep3	190124	3852	148
	yVW416	0.2M NaCl	rep1	181207	3796	175
**Figure 2 g**	yVW403	0.2M NaCl Glucose	B	190625	4076	248
	yVW403	0.2M NaCl Raffinose	B	190625	4076	275
**Figure 3 a middle**	ySP329	0.1M NaCl	C	190412	3989	327
	ySP329	0.2M NaCl	C	190412	3989	341
	ySP329	0.3M NaCl	C	190412	3989	311
**Figure 3 a bottom**	Data from Figure 1d					
**Figure 3 b**	Data from Figure 2a					
**Figure 3 c**	Data from Figure 1d					
**Figure 4a**	Data from Figure 2a					
**Figure 4 b**	yVW430	0.0 M NaCl	rep1	181127	3756	248
	yVW429	0.0 M NaCl	rep1	190222	3873	216
	yVW432	0.0 M NaCl	rep1	181120	3743	214
**Figure 4 c**	yVW405	0.2M NaCl	rep3	181129	3772	349
	yVW407	0.2M NaCl	rep1	181120	3742	529
	yVW471	0.2M NaCl	rep1	190322	3920	293
	yVW472	0.2M NaCl	rep3	190405	3968	297
**Figure 5**	Data from Figure 2a					
**Figure 6 a**	Data from Figure 1d					
**Figure 6 c**	yVW474	Pulse	D	190619	4056	168
	yVW474	Step	D	190619	4054	159
	yVW474	Ramp	D	190619	4052	119
**Figure 7 a**	Data from Figure 2a					
**Sup Fig 1**	ySP884	0.0 M NaCl	B	190625	4074	266
	ySP884	0.1M NaCl	B	190625	4074	370
	ySP884	0.2M NaCl	B	190625	4074	396
	ySP884	0.3M NaCl	B	190625	4074	291
**Sup Fig 4**	yVW403	0.2M NaCl	B	200117	4205	436
	ySP929	0.2M NaCl	A	200117	4200	408

	ySP930	0.2M NaCl	B	200117	4203	483
**Sup Fig 5**	yVW474	0.0 M NaCl	rep2	190503	4009	65
	yVW474	0.1M NaCl	rep2	190503	4010	143
	yVW474	0.2M NaCl	rep2	190503	4009	195
	yVW474	0.3M NaCl	rep2	190503	4010	306
**Sup Fig 6**	ySP927	0.2M NaCl	A	191219	4191	89
	ySP927	0.2M NaCl	B	191219	4192	91
	ySP927	0.2M NaCl	C	191219	4193	77
**Sup Fig 8**	yVW431	0.0 M NaCl	rep2	181129	3769	183
	yVW428	0.0 M NaCl	rep2	181213	3820	120
	yVW403	0.0 M NaCl	rep1	181120	3739	313
	yVW430	0.0 M NaCl	rep1	181127	3756	248
	yVW429	0.0 M NaCl	rep1	190222	3873	216
	yVW432	0.0 M NaCl	rep1	181120	3743	214
**Sup Fig 9 a**	Data from Figure 3a					
**Sup Fig 9 b**	Data from Figure 1d					
**Sup Fig 9 c**	Data from Figure 2a					
**Sup Fig 10 a**	Data from Figure 2a					
**Sup Fig 10 b**	Data from Figure 4b					
**Sup Fig 12**	yVW403	0.2M NaCl	rep1	191206	4164	269
	ySP915	0.2M NaCl	B	191206	4166	282
	yVW432	0.2M NaCl	rep1	191206	4168	293
	ySP918	0.2M NaCl	A	191206	4169	310
**Sup Fig 13**	yVW474	0.2M NaCl	C	191217	4187	366
	ySP919	0.2M NaCl	E	191217	4188	257
	ySP899	0.2M NaCl	B	191217	4189	319
	ySP922	0.2M NaCl	D	191217	4190	367
**Sup Fig 14**	Data from Figure 2a					

Supplementary Figures

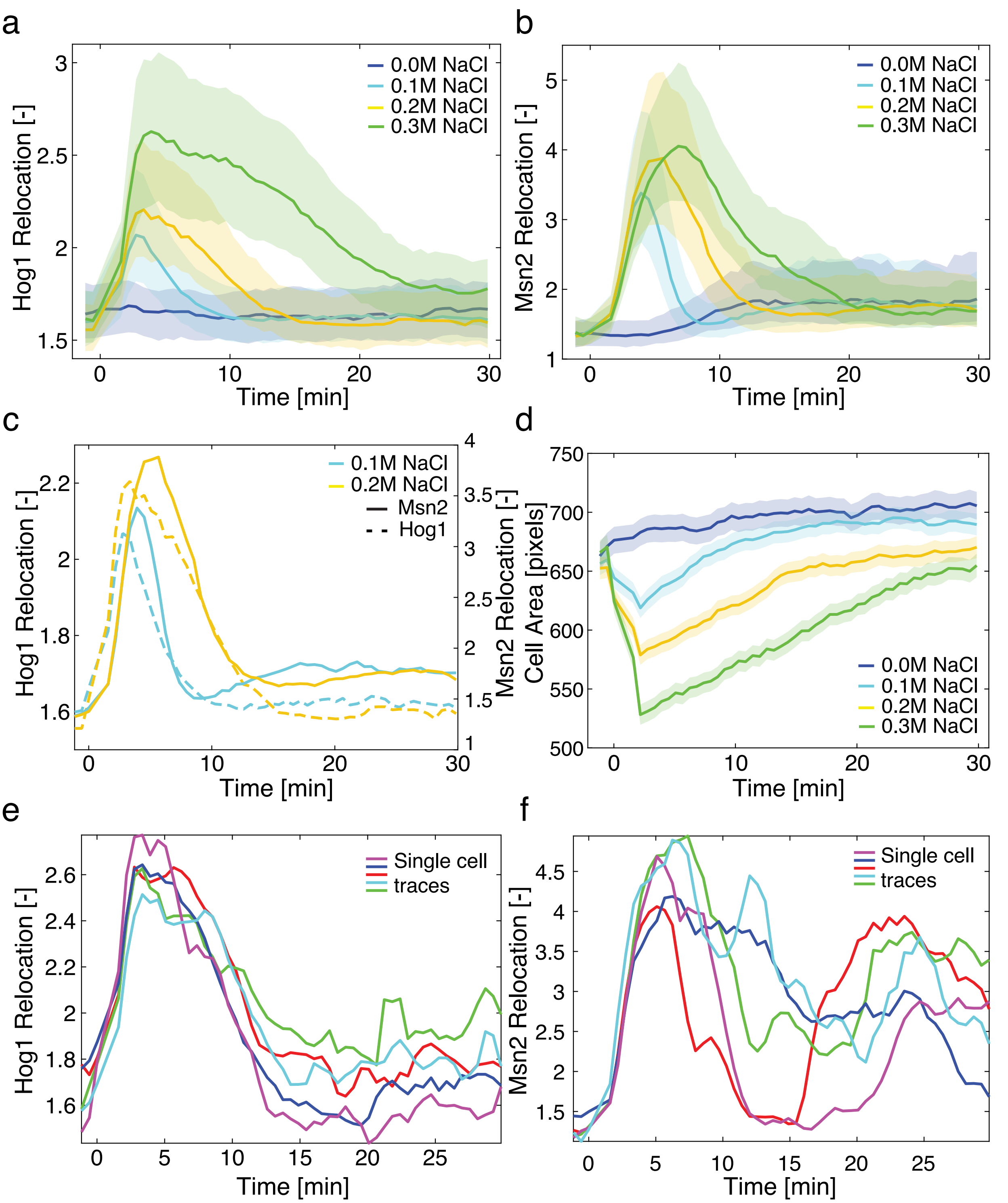

Supplementary Figure 1. Nuclear relocation of Msn2 and Hog1.
a. - c. Strains bearing a Hta2-CFP, Hog1-mCitrine and Msn2-mCherry were stressed with various concentrations of NaCl. The nuclear relocation was quantified by the ratio in nuclear over cytoplasmic fluorescence. The median (solid line) and 25th -75th percentiles (shaded areas) are plotted for Hog1 in the yellow channel (a), Msn2 in the red channel (b) and directly compared on the same graph (c) for at least 260 cells. **d.** Change in cell size following hyper-osmotic stresses. The median (solid line) and 25th -75th percentiles (shaded areas) are displayed. **e. - f.** The nuclear relocation traces from the same single cells for Hog1 (e) and Msn2 (f) are plotted. These cells were selected in the population because they display a strong re-entry of Msn2 in the nucleus following the first pulse of activity. This second phase in the response is absent from the Hog1 dynamics.

a

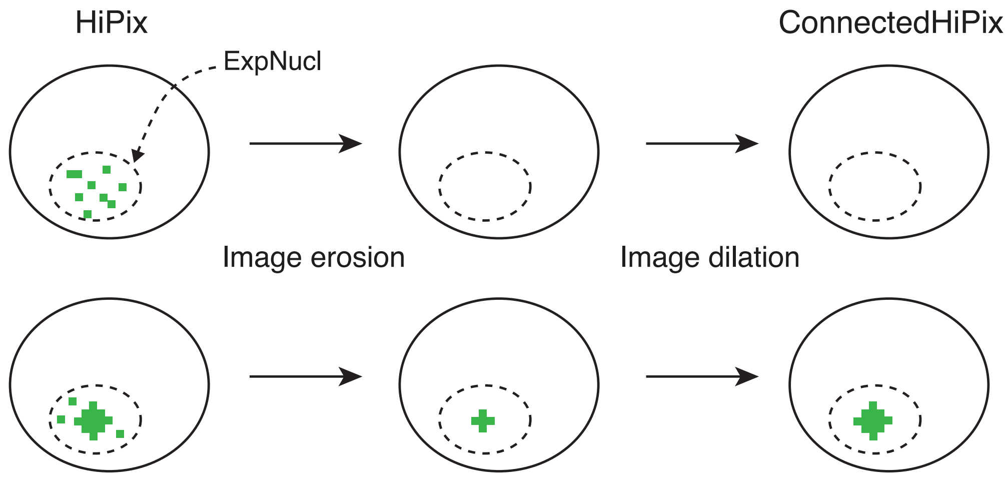

b

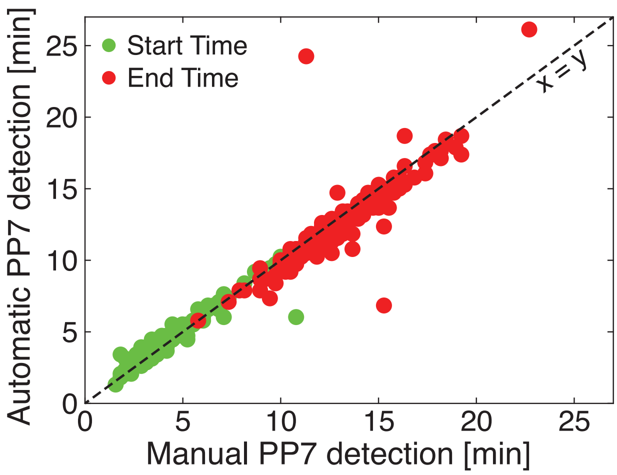

c

		Manual Curation	
		**PP7 Positive**	**PP7 Negative**
**Automated measurement**	**PP7 Positive**	193	1
	**PP7 Negative**	1	43

Supplementary Figure 2. Quantification of the PP7 traces with the ConnectedHiPix feature.

a. Scheme describing the process used to generate the ConnectedHiPix feature from the 20 highest intensity pixels (HiPix) in the ExpNucl object (Nucleus expanded by 5 pixels). **b.** Comparison between a manual curation of Start Times (green) and End Times (red) by visual inspection of the cells and automated quantification by the ConnectedHiPix measurement. **c.** Table displaying the number of PP7 positive and negative cells obtained by manual versus automated quantification.

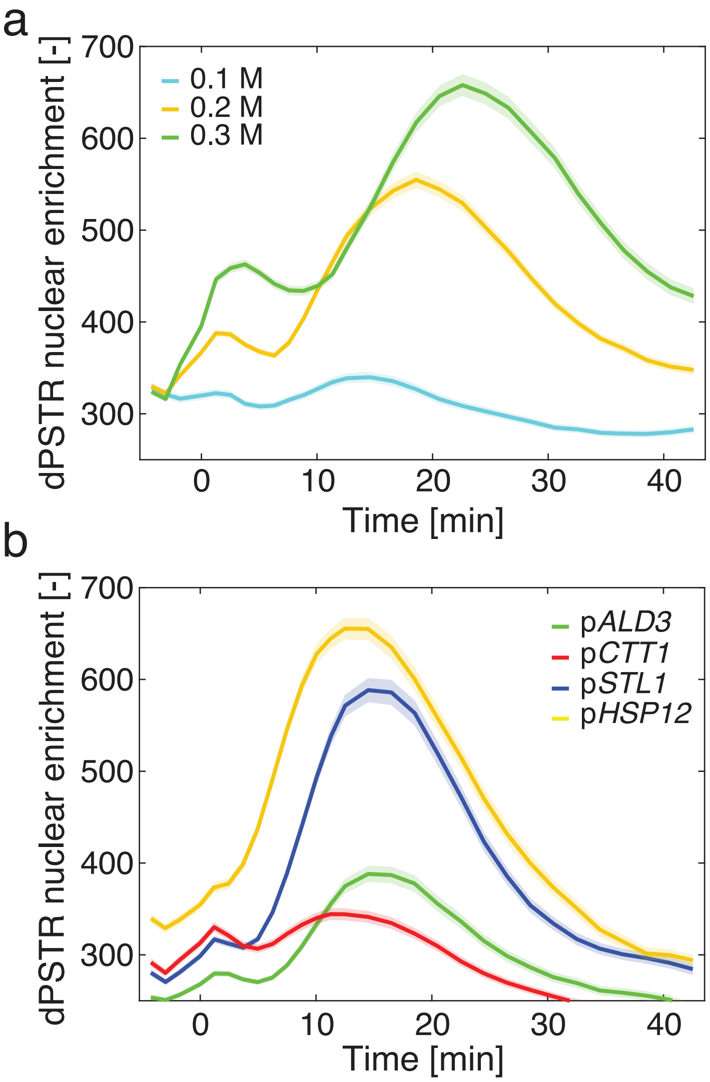

Supplementary Figure 3. Dynamic protein synthesis translocation reporter (dPSTR) induction upon osmotic stress.

The dPSTR reporter allows to by-pass the slow maturation time of protein expression reporters. A constitutively expressed FP is functionalized by a leucine zipper. The compatible zipper is under the control of the inducible promoter of interest and coupled to two strong Nuclear Localization Signals (NLS) motifs. When the pair of leucine zippers interact, the enrichment of the FP in the nucleus allows to quantify the level of induction of the promoter[5]. **a.** p*STL1*-dPSTR^R nuclear enrichment (nuclear fluorescence minus cytoplasmic fluorescence) for three different stress levels. **b.** dPSTR^R nuclear enrichment for 4 different promoters following a 0.2M NaCl stress. In all graphs, the solid lines represent the mean responses of a population of more than 400 cells and the shaded areas represent the s.e.m..

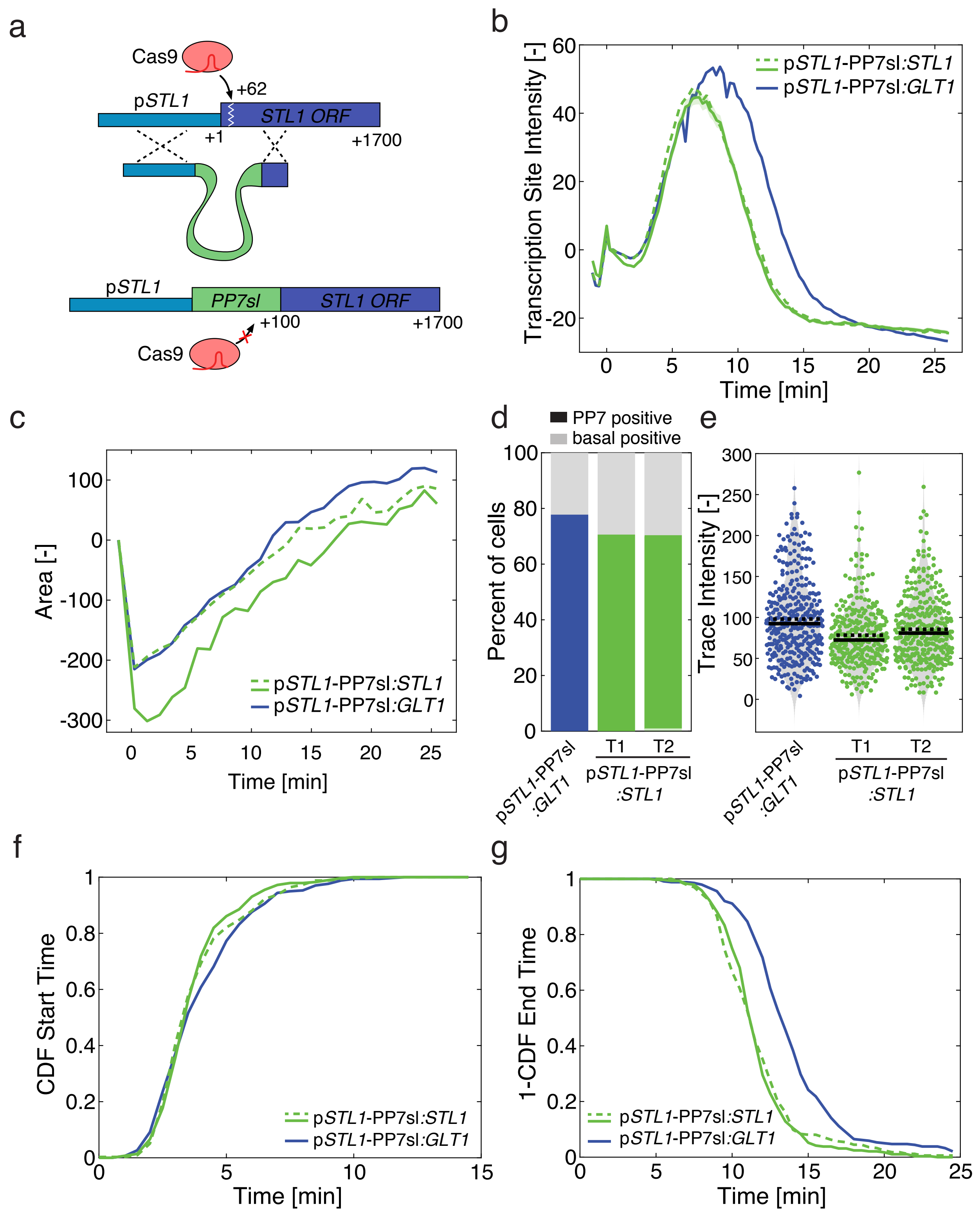

Supplementary Figure 4. Monitoring transcription from the endogenous *STL1* locus
a. Scheme describing the integration of the 24xPP7sl at the *STL1* locus by CRISPR-Cas9. The sgRNA recognizes the PAM motif 62bp upstream of the start codon. The DNA break is repaired by homologous recombination from a DNA fragment that contains homology with the *STL1* promoter and 500 bp from the *STL1* ORF starting at position 100. **b.** Transcription site intensity arising from the p*STL1* promoter upon 0.2M NaCl stress. **c.** Cell size adaptation dynamics following hyper-osmotic for the experiment presented in b. **d.** Percent of PP7 positive cells. **e.** Maximum intensity of single-cell traces. **f.** and **g.** Dynamics of transcription onset and shut off represented with the CDF of Start Times (f) and 1-CDF of End Times (g). In all these graphs, the response arising from the *GLT1* locus (blue) is compared to the one monitored at the native *STL1* locus (green). Two different transformants were measured in order to verify that undesired Cas9 activity has not disrupted the HOG response.

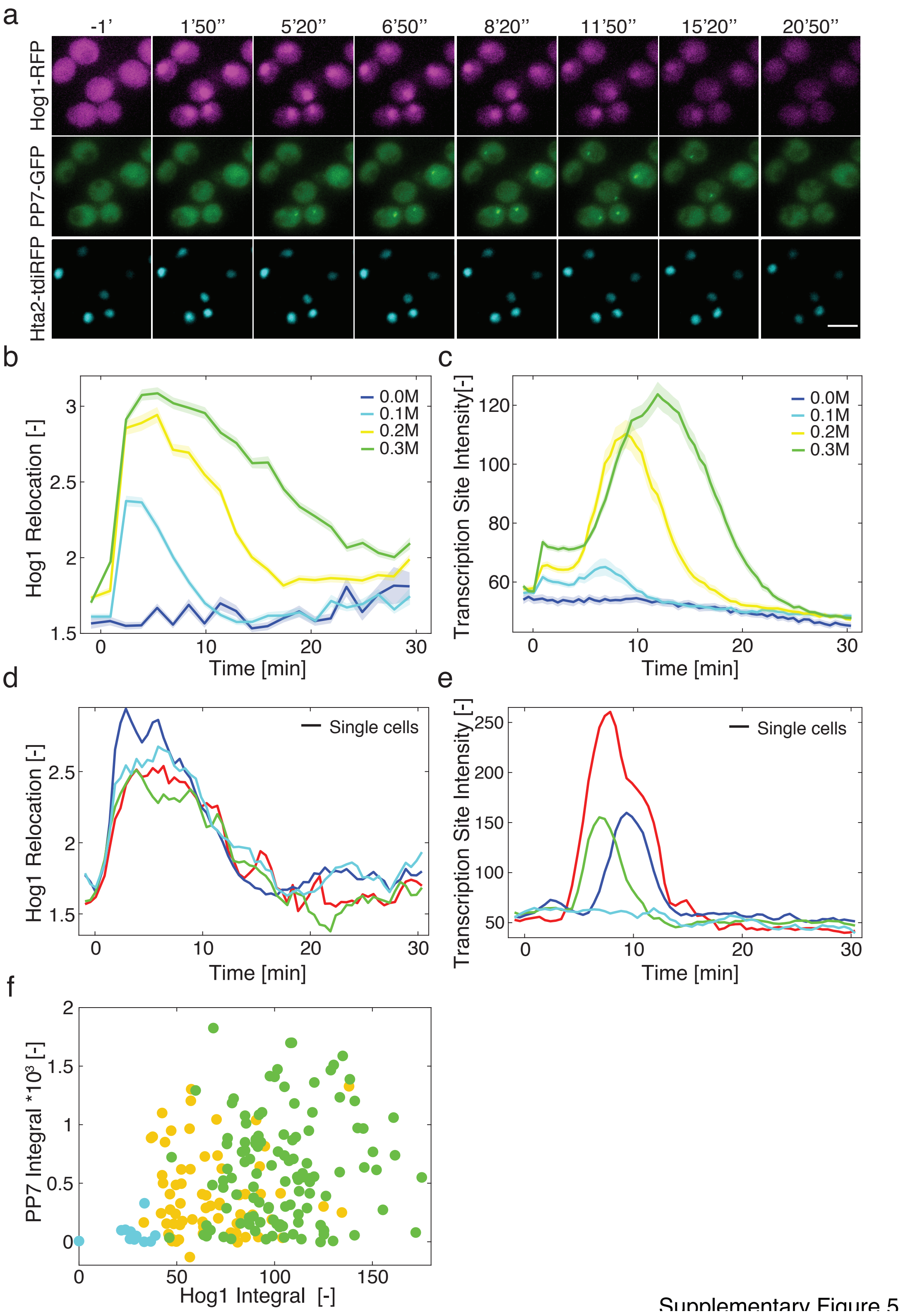

Supplementary Figure 5. Correlating Hog1 activity and downstream transcription in the same cell.

a. Thumbnails images of the strain combining a Hta2-tdiRFP nuclear marker, Hog1-mCherry and the p*STL1*-PP7 reporter. Cells were stressed with 0.2M NaCl at time 0. Scale bar is 5 μm. Representative images from at least three biological replicates. **b. - c.** Mean dynamics of Hog1 nuclear enrichment (b) and PP7 transcription site fluorescence intensity (c) following different osmotic stresses. More than 140 cells are quantified for the inducing conditions and only 65 in the SD-full experiment. The solid lines represent the mean response and the shaded areas represent the s.e.m.. **d. - e.** Examples of single-cell traces that display similar Hog1 relocation dynamics (d) and different transcriptional responses as quantified by the p*STL1*-PP7 transcription site intensity (e). **f.** Correlation between Hog1 relocation and the PP7 output measured by their integrals. Each dot corresponds to a single-cell measurement.

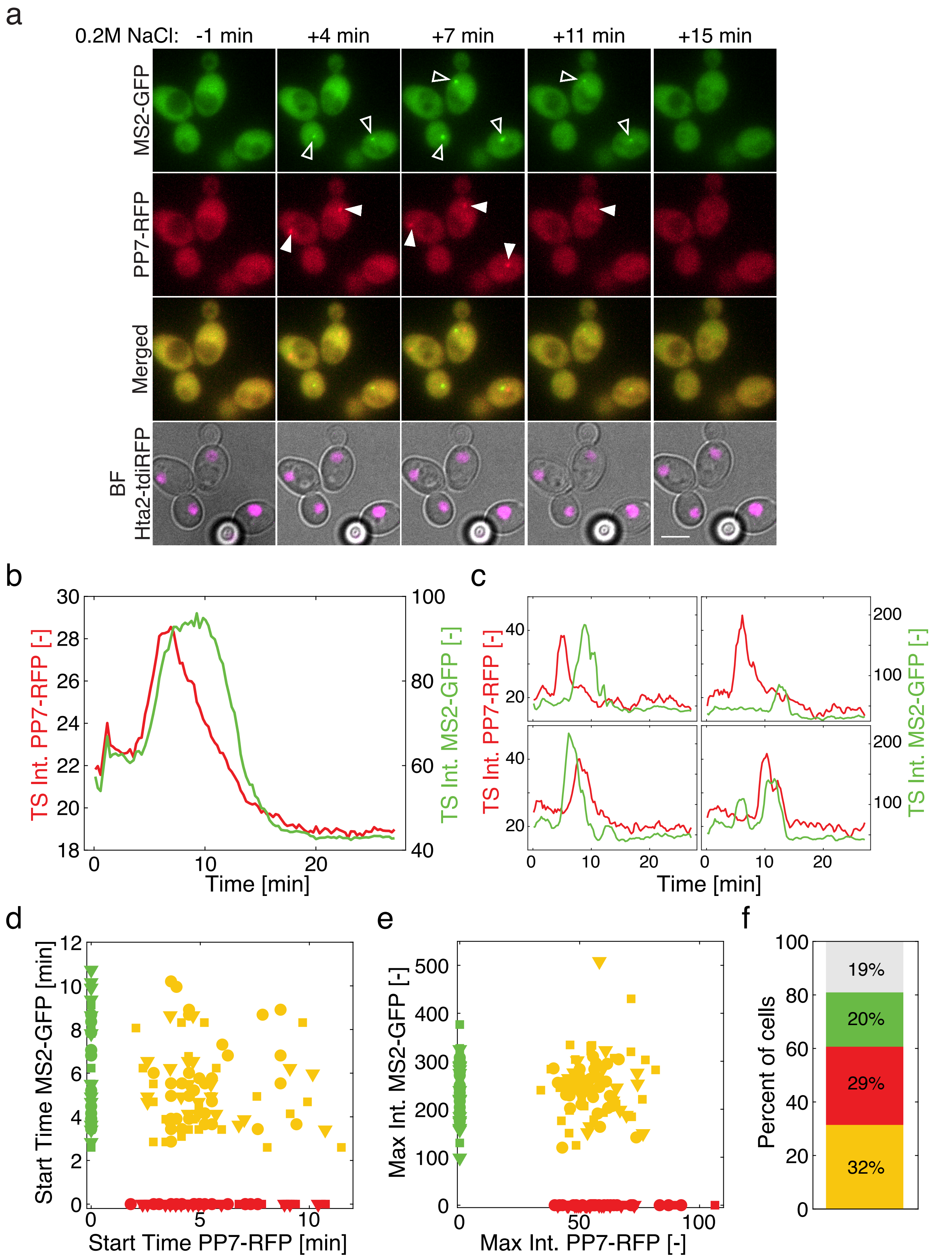

Supplementary Figure 6. Monitoring p*STL1*-induced transcription from two identical loci in diploids.

a. Thumbnails of diploid cells bearing the MS2-GFP and PP7-mCherry reporter systems monitoring the induction of two p*STL1* in the same cell following 0.2M NaCl stress. Open arrowheads (MS2sl) and closed arrowheads (PP7sl) highlight the stochastic activation of the transcription within a cell.

Scale bar 5μm. Representative images from at least three biological replicates. **b.** Transcription site intensity from the p*STL1* promoter monitored with the MS2 (green) or the PP7 (red) systems. The low signal provided by the PP7-mCherry assay and bleaching of this FP can explain the discrepancy between the two reporter systems. **c.** Examples of single cell traces where the activation of both *STL1* promoters was detected. **d.** and **e.** Scatter plots representing the Start Times (d) and the Maximum Intensity (e) of the PP7 and MS2 assays. The data from three different experiments (rounds, squares and triangles) are combined. Cells where only the MS2 system activation was detected are plotted in green, while cells with only PP7 TS are in red. Cells where both systems where detected are in yellow. **f.** Mean percentage of cells over the three experiments where both promoters (yellow) or only one promoter (green / MS2 and red / PP7) or no transcription (gray) was detected.

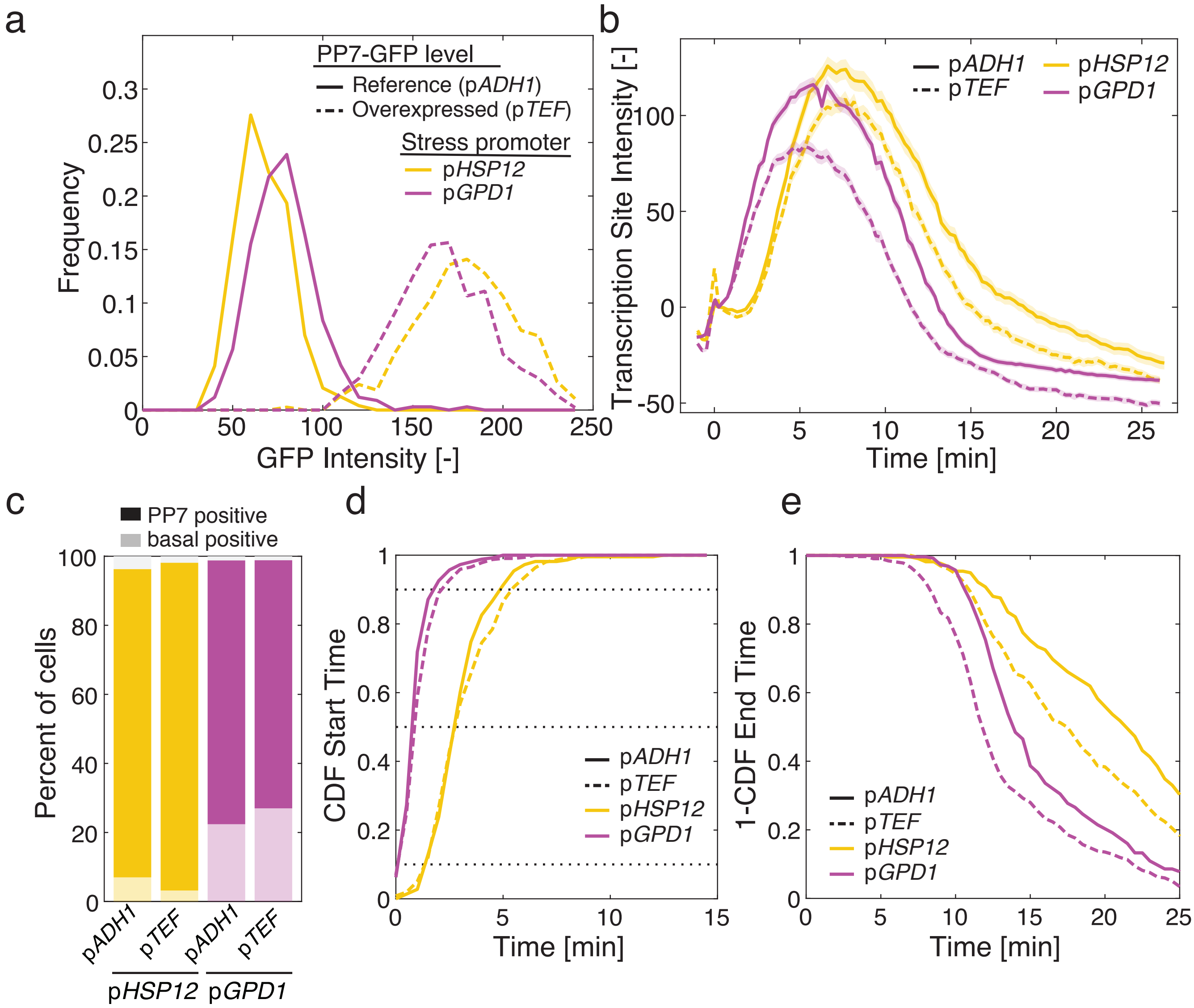

Supplementary Figure 7. Testing the effect of the overexpression of PP7-GFP on the transcription site measurements.

a. Comparison of the initial GFP fluorescence for the PP7-GFPenvy expressed from the p*ADH1* (reference) or p*TEF* promoter (overexpression) which leads to a 3-fold higher fluorescence. **b.** Mean transcription site intensity following a 0.2M NaCl stress. The lines represent the mean response of the population and the shaded areas represent the s.e.m.. **c.** Percentages of PP7 positive cells. Cells displaying a TS before time point 4 (basal positive) are displayed in a lighter color. **d.** Cumulative distribution of Start Times. **e.** One minus the cumulative distribution function of End Times. The lower expression level of the PP7-GFP by the reference p*ADH1* promoter improves the detection efficiency of the TS.

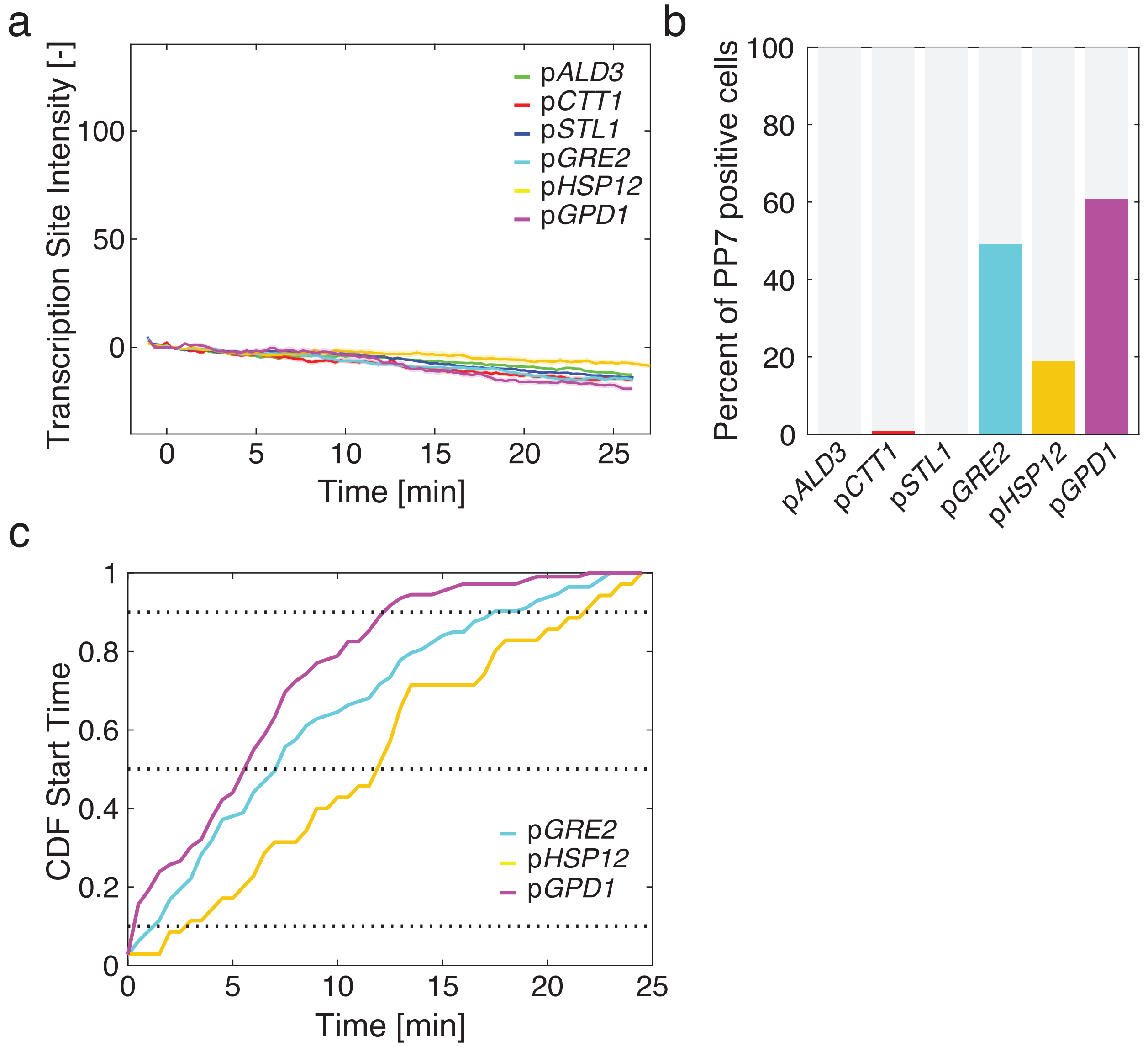

Supplementary Figure 8. HOG promoters with basal expression level.
a. Average transcription site intensity following an SD-full addition. The solid lines represent the mean ratio and the shaded areas represent the s.e.m. of at least 120 cells. **b.** Percentages of PP7 positive cells during the entire SD-full time-lapse experiment in a. **c.** Cumulative distribution function of Start Times for the promoters displaying more than 10% of expressing cells in the SD-full time-lapse experiment.

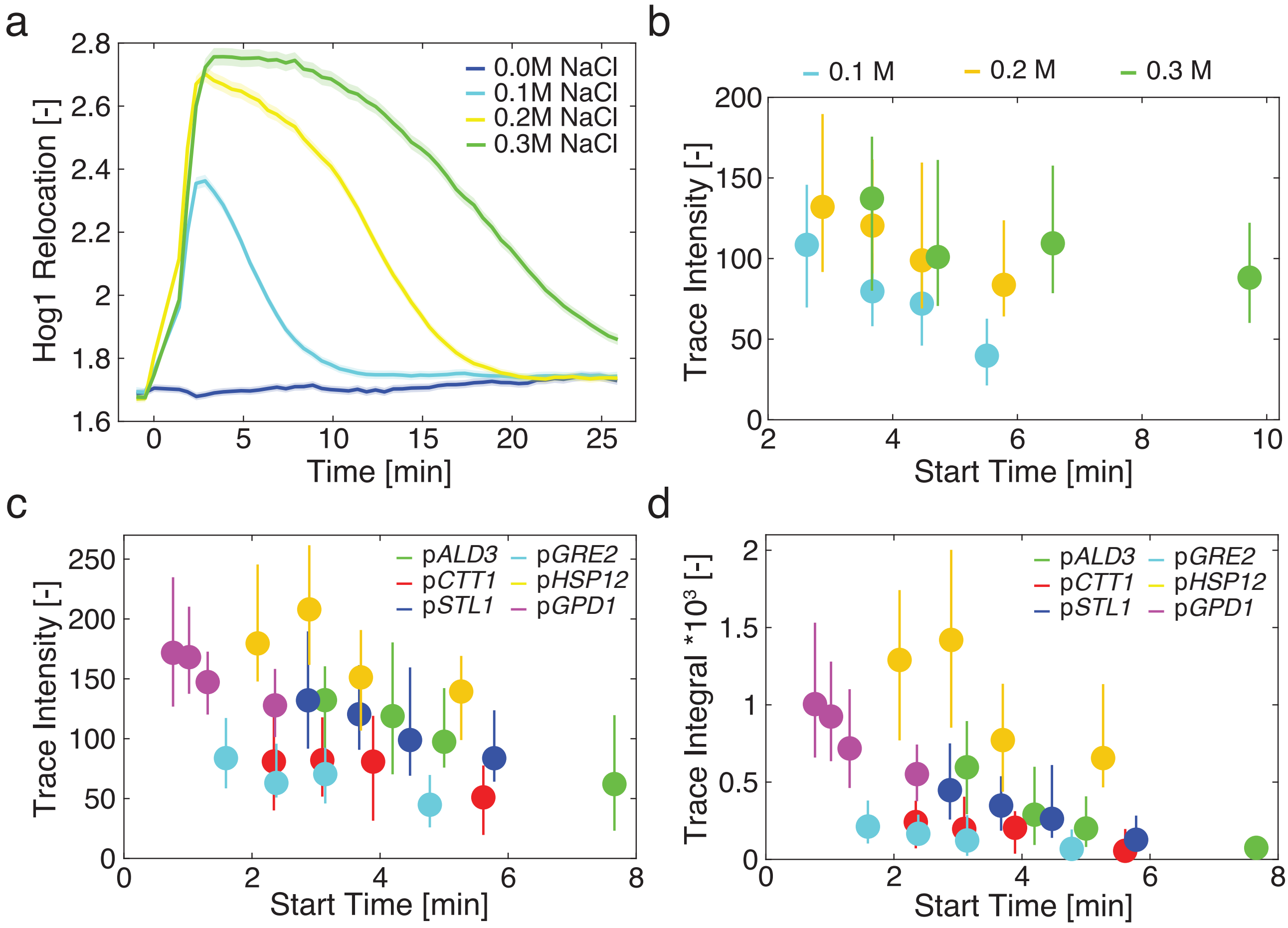

Supplementary Figure 9. Negative correlation between Start Time and transcriptional output for all HOG promoters.

a. Dynamics of Hog1 nuclear enrichment following hyper-osmotic stress. The mean ratio of nuclear over cytoplasmic fluorescence of Hog1-GFP for more than 250 cells is plotted as function of time. The shaded area represents the s.e.m. **b.** The population of p*STL1*-PP7 positive cells is split in four quartiles (of at least 50 cells each) based on their Start Time. The median (circle) and 25th to 75th percentiles (line) of the intensity of the PP7 trace is plotted for each quartile. **c.- d.** Plot of the Start Time versus the Trace intensity (c) or Trace integral (d) for all the PP7 reporter strains following a 0.2M NaCl stress. The population of responding cells is split in four quartiles (of at least 35 cells each) based on their Start Time. The median (circle) and 25th to 75th percentiles (line) of the Trace Intensity (c) or Trace Integral (d) are plotted for each quartile.

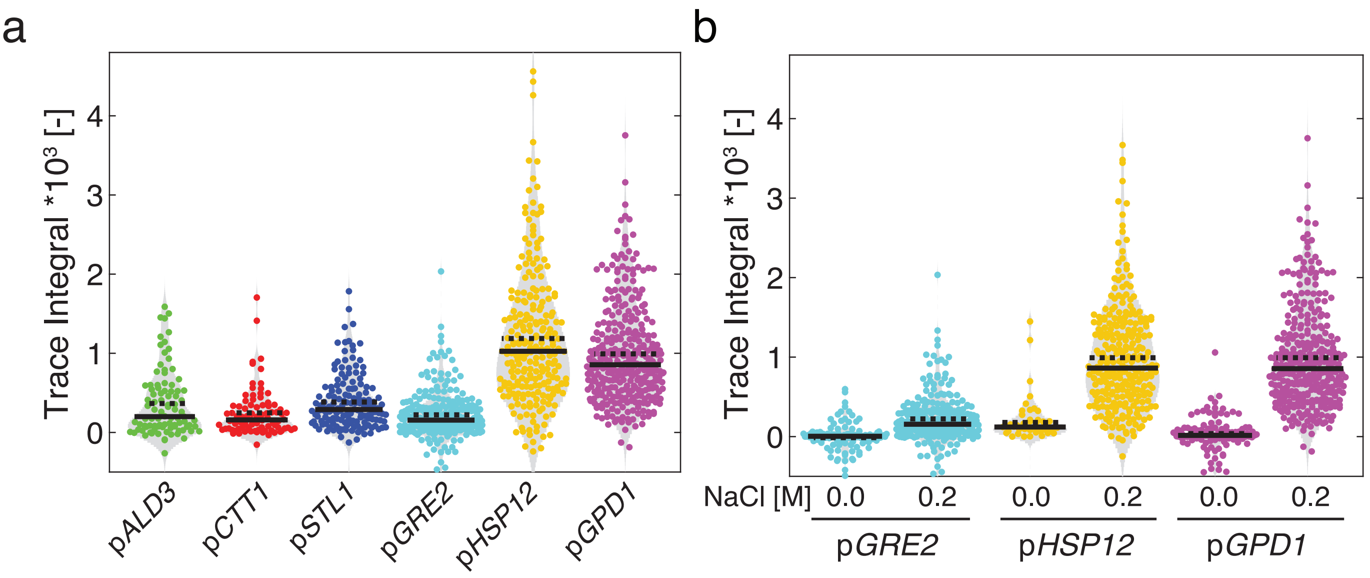

Supplementary Figure 10. Trace integral of HOG promoters.
a. Violin plots of the Trace integral of osmostress promoters response after 0.2M NaCl treatment.
b. Violin plots of the Trace integral of basal level positive osmostress promoters response after SD-full (0.0M) or 0.2M NaCl treatment. For both graphs, each dot represents the data of a single cell, the full line the median of the response and the dashed line the mean of the response.

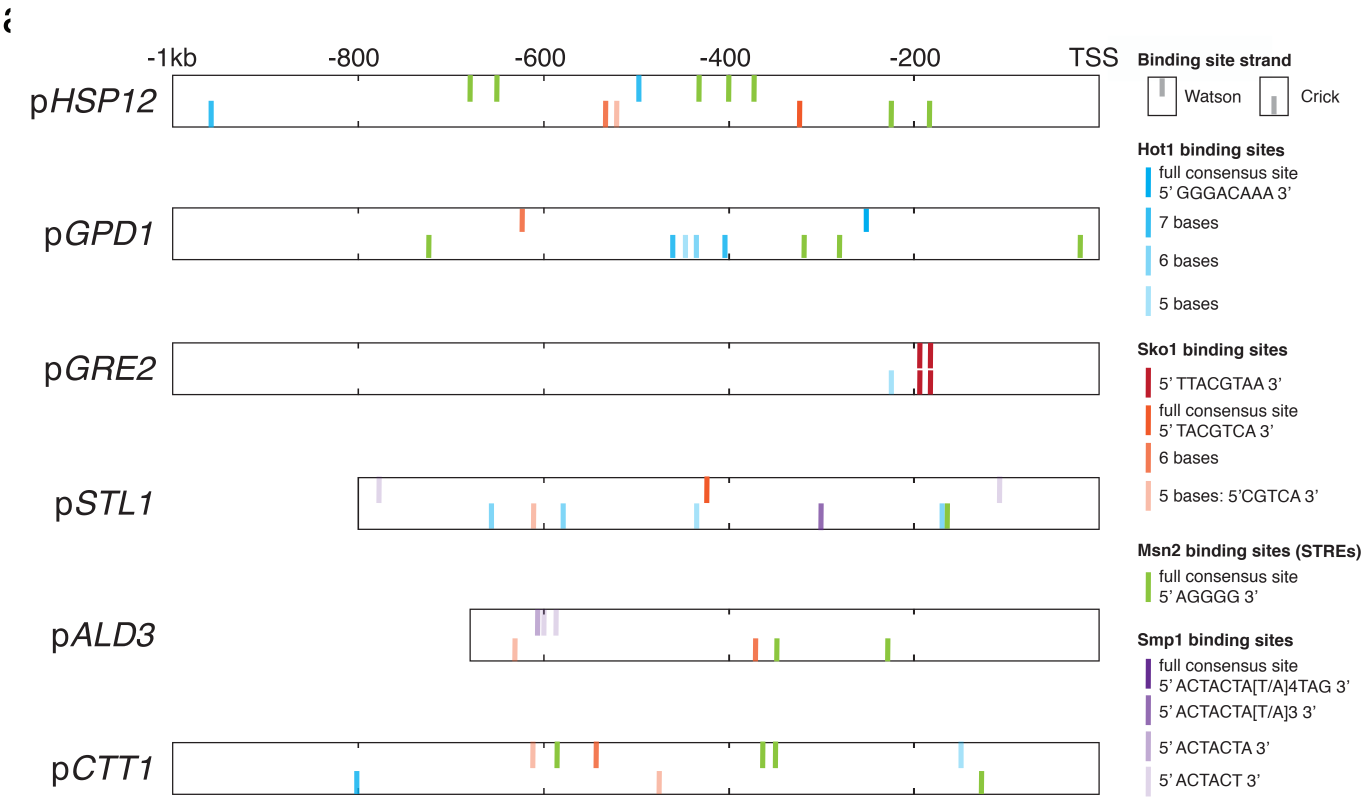

Supplementary Figure 11. Stress promoter architecture.
Consensus binding sites of Hot1[6], Sko1[7], Msn2/4[8], and Smp1[9] and some deviations from these consensus sequences have been mapped on the six promoters used in this study.

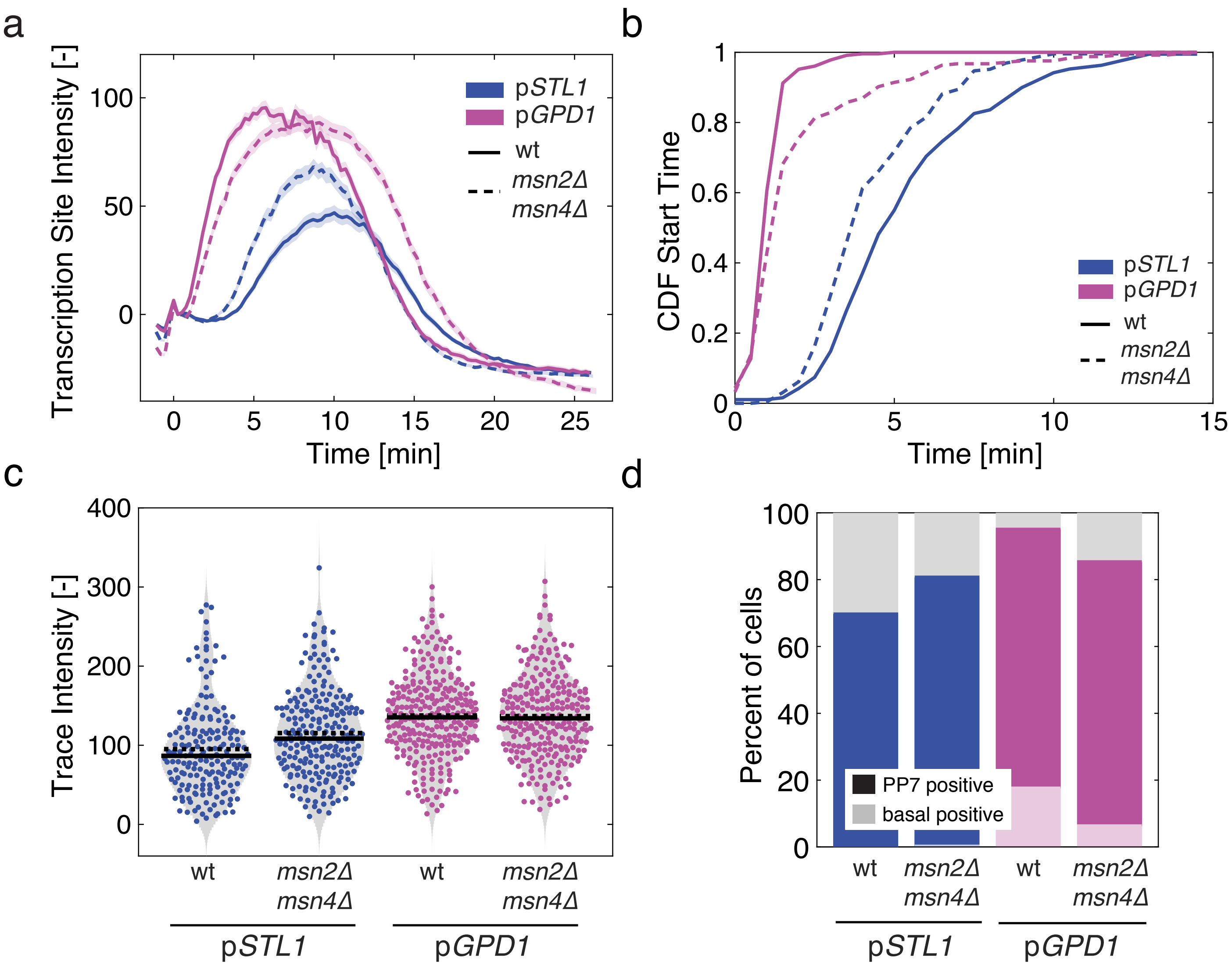

Supplementary Figure 12. Analysis of transcription dynamics in the *msn2Δmsn4Δ* mutant

a. Transcription site intensity of WT (solid line) and *msn2Δmsn4Δ* (dashed line) bearing the p*STL1*-PP7 (blue) or the p*GPD1*-PP7 (magenta) reporters following a 0.2M NaCl stress. The lines represent the mean response of the population and the shaded areas represent the s.e.m.. **b.** Cumulative distribution of Start Time for the two promoters in the WT and mutant backgrounds for cells that induce transcription after time zero. **c.** Violin plots of the trace intensity (maximum of the TS during the transcription period) after stimulation by 0.2M NaCl. Each dot represents the value calculated from a single cell. The lines represent the median and the dashed line the mean of the population. **d.** Percentages of cells where a PP7 TS site was detected. The light shaded area represents the percentage of PP7 positive cells before the stimulus was added (basal transcription).

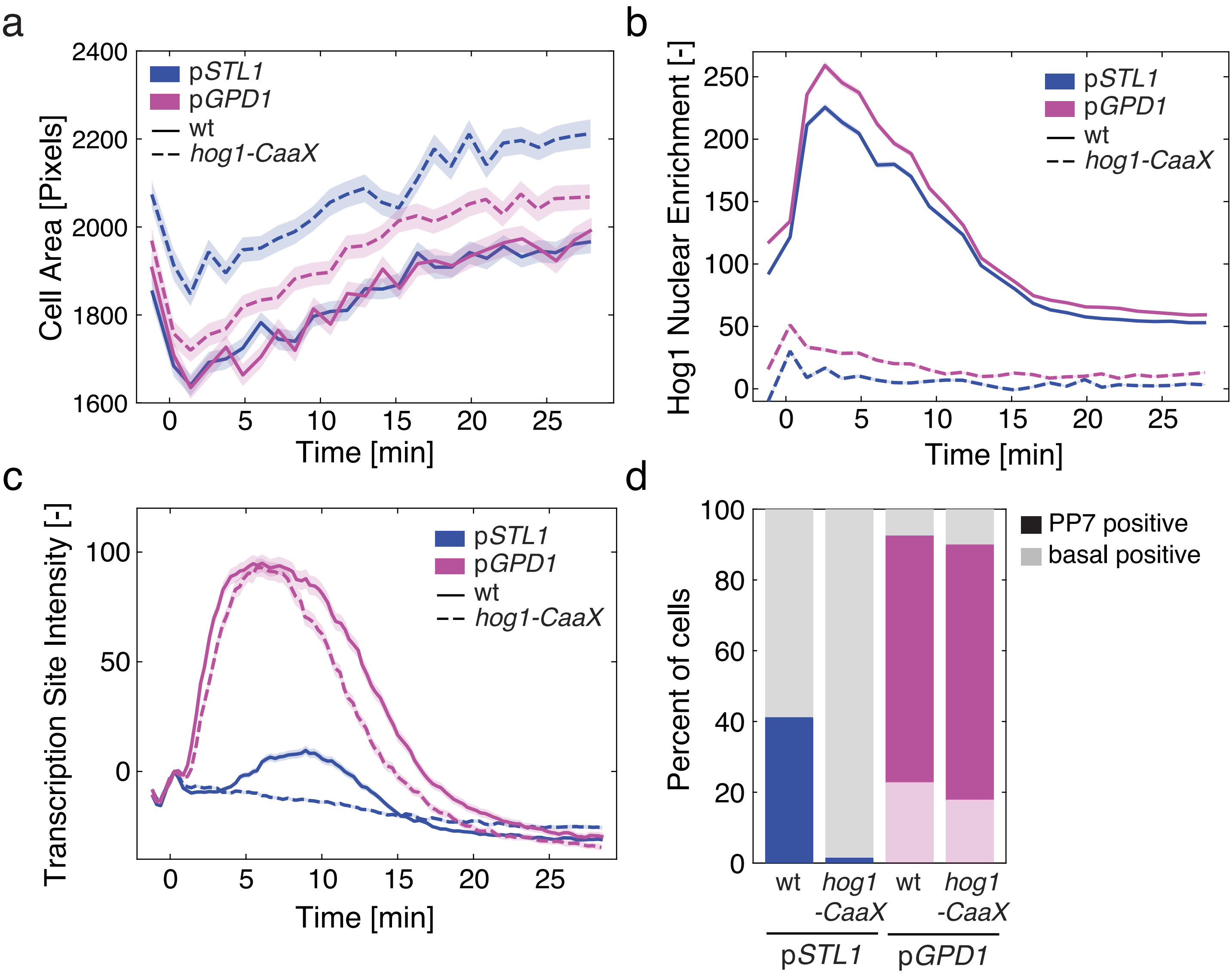

Supplementary Figure 13. Impact of Hog1 anchoring at the plasma membrane of p*STL1* and p*GPD1* activation.

a. - c. Dynamics of cell size adaptation (a), Hog1-mCherry nuclear enrichment (b) and Transcription Site intensity (c) in cells expressing either freely diffusing (wt, solid line) or membrane anchored Hog1 via a CaaX motif (dashed line) for the two transcriptional reporters p*STL1*-PP7 (blue) and p*GPD1*-PP7 (magenta) upon 0.2M NaCl stress. The lines represent the mean response of the population and the shaded area represents the s.e.m.. **d.** Percentage of cells where a PP7 TS site was detected. The light shaded area represents the percentage of PP7 positive cells before the stimulus was added (basal transcription).

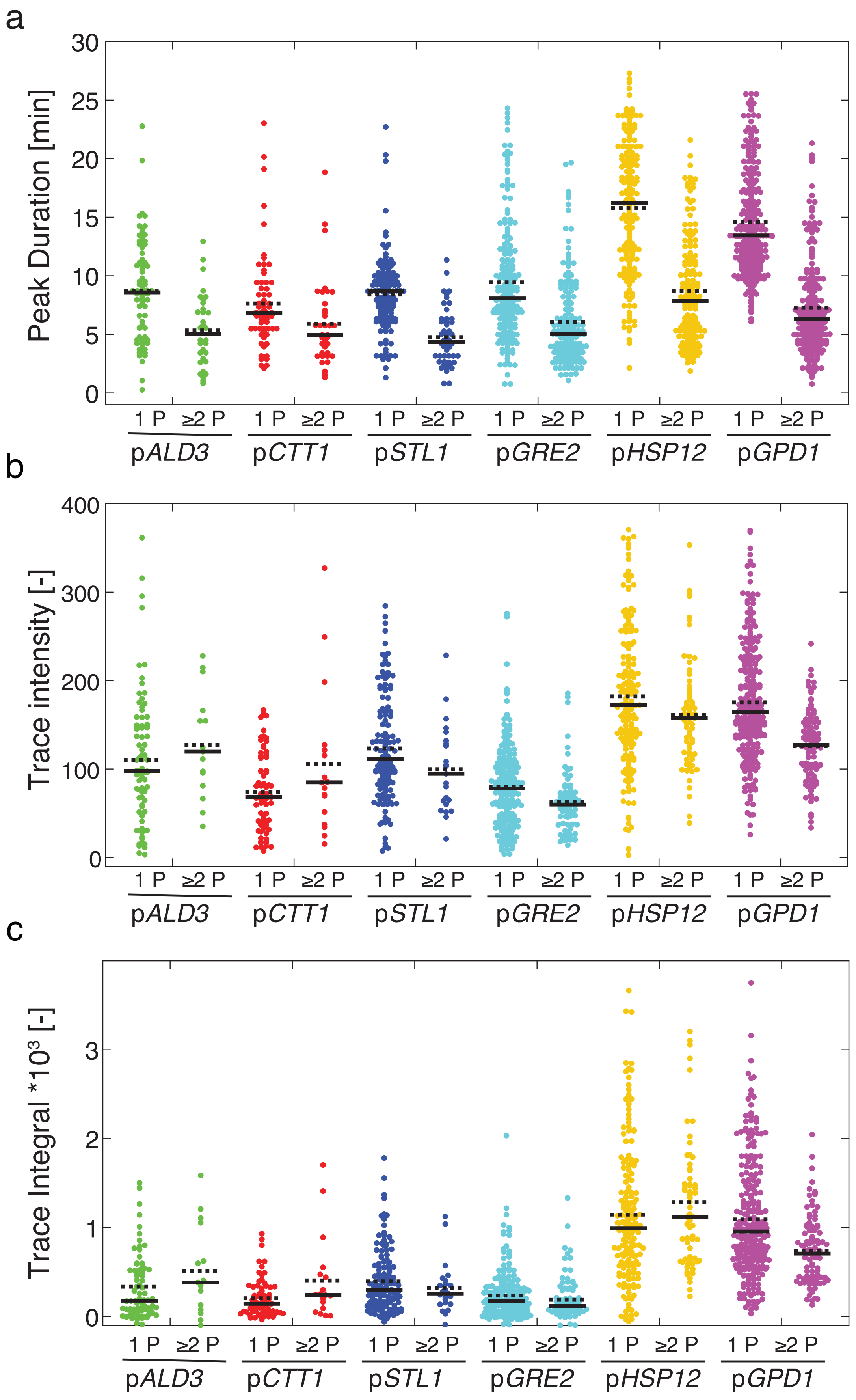

Supplementary Figure 14. Peak analysis of osmostress-promoter in response to 0.2M NaCl.

a. - c. Violin plots of the Peak Duration (a), Trace Intensity (b) and Trace Integral (c) for the different PP7 reporter strains stresses with 0.2 M NaCl. The population of cells was split between cells displaying one peak and cells where two peaks or more were detected. Each dot represents the value calculated for a single peak (a) or single cell (b and c). The solid line is the median and the dashed line the mean of the population.

Supplementary References

1. Larson, D. R., Zenklusen, D., Wu, B., Chao, J. A. & Singer, R. H. Real-Time Observation of Transcription Initiation and Elongation on an Endogenous Yeast Gene. *Science* **332**, 475–478 (2011).
2. Wosika, V. *et al.* New families of single integration vectors and gene tagging plasmids for genetic manipulations in budding yeast. *Molecular Genetics and Genomics* **291**, 2231–2240 (2016).
3. Laughery, M. F. *et al.* New vectors for simple and streamlined CRISPR-Cas9 genome editing in Saccharomyces cerevisiae. *Yeast* **32**, 711–720 (2015).
4. Ralser, M. *et al.* The Saccharomyces cerevisiae W303-K6001 cross-platform genome sequence: insights into ancestry and physiology of a laboratory mutt. *Open Biol* **2**, 120093 (2012).
5. Aymoz, D., Wosika, V., Durandau, E. & Pelet, S. Real-time quantification of protein expression at the single-cell level via dynamic protein synthesis translocation reporters. *Nature Communications* **7**, 11304 (2016).
6. Gomar-Alba, M., Alepuz, P. & del Olmo, M. lí. Dissection of the elements of osmotic stress response transcription factor Hot1 involved in the interaction with MAPK Hog1 and in the activation of transcription. *Biochim Biophys Acta* **1829**, 1111–1125 (2013).
7. Proft, M., Gibbons, F. D., Copeland, M., Roth, F. P. & Struhl, K. Genomewide identification of Sko1 target promoters reveals a regulatory network that operates in response to osmotic stress in Saccharomyces cerevisiae. *Eukaryotic Cell* **4**, 1343–1352 (2005).
8. Martínez-Pastor, M. T. *et al.* The Saccharomyces cerevisiae zinc finger proteins Msn2p and Msn4p are required for transcriptional induction through the stress response element (STRE). *EMBO J* **15**, 2227–2235 (1996).
9. Dodou, E. & Treisman, R. The Saccharomyces cerevisiae MADS-box transcription factor Rlm1 is a target for the Mpk1 mitogen-activated protein kinase pathway. *Mol Cell Biol* **17**, 1848–1859 (1997).
