# Supplementary material for: Single-particle imaging of stress-promoters induction reveals the interplay between MAPK signaling, chromatin and transcription factors
Source: Nat Commun. 2020 Jun 23;11:3171. doi: 10.1038/s41467-020-16943-w (PMC7311541; doi:10.1038/s41467-020-16943-w)
Supplement: Supplementary file 3 — Description of Additional Supplementary Information [file 41467_2020_16943_MOESM3_ESM.pdf]

Description of Additional Supplementary Files

File Name: Supplementary Movie 1
Description: Time lapse movie of the pSTL1-PP7 reporter strain. The left image is a maximum intensity projection of a Z-stack in the green channel allowing to image the PP7-GFP and visualizing the presence of transcription sites as bright foci. The central image is the red channel image, representing the fluorescence of the Hta2-mCherry nuclear tag. The right image is a merged image between the green and red channels. The number in the upper right corner indicates the time in minutes. Cells are stressed with 0.2M NaCl at time 0.

File Name: Supplementary Movie 2
Description: Time lapse movie of a diploid pSTL1-PP7 / pSTL1- MS2 reporter strain. The leftmost image is a maximum intensity projection of a Z-stack in the green channel allowing to image the MS2-GFP and visualizing the presence of transcription sites as bright foci. The center-left image is a maximum intensity projection of a Z-stack in the red channel allowing to image the PP7-mCherry and visualize the presence of transcription sites as bright foci. The center-right image is the far-red channel image representing the fluorescence of the Hta2-tdiRFP nuclear tag. The rightmost image is a merged image between the green and red channels (PP7 and MS2) allowing to observe to which extent the induction of two pSTL1 correlate in the same cell. The number in the lower right corner indicates the time in minutes. Cells are stressed with 0.2M NaCl at time 0.

File Name: Supplementary Movie 3
Description: Time lapse movie of the pGPD1-PP7 reporter strain. The left image is a maximum intensity projection of a Z-stack in the green channel allowing to image the PP7-GFP and visualizing the presence of transcription sites as bright foci. Note the presence of some transcription sites in absence of stimulus in the first frames of the movie. The central image is the red channel image, representing the fluorescence of the Hta2-mCherry nuclear tag. The right image is a merged image between the green and red channels. The number in the upper right corner indicates the time in minutes. Cells are stressed with 0.2M NaCl at time 0.

File Name: Supplementary Movie 4
Description: Time lapse movie of the pSTL1-PP7 in a step experiment. The leftmost image is a maximum intensity projection of a Z-stack in the green channel allowing to image the PP7-GFP and visualize the presence of transcription sites as bright foci. The background fluorescence in the image allows to follow NaCl concentration changes in the flow channel. Higher fluorescence is indicative of lower NaCl concentrations. The center-left image is the red channel image and allows to follow the changes in Hog1 nuclear localization. The center-right image is the far-red channel image representing the fluorescence of the Hta2-tdiRFP nuclear tag. The rightmost image is a merged image between the green and red channels (PP7 and Hog1). The number in the lower right corner indicates the time in minutes from the start of the experiment.

File Name: Supplementary Movie 5
Description: Time lapse movie of the pSTL1-PP7 in a pulse experiment. The leftmost image is a maximum intensity projection of a Z-stack in the green channel allowing to image the PP7-GFP and visualizing the presence of transcription sites as bright foci. The background fluorescence in the image allows to follow NaCl concentration changes in the flow channel. Higher fluorescence is

indicative of lower NaCl concentrations. The center-left image is the red channel image and allows to follow the changes in Hog1 nuclear localization. The centerright image is the far-red channel image representing the fluorescence of the Hta2- tdiRFP nuclear tag. The rightmost image is a merged image between the green and red channels (PP7 and Hog1). The number in the upper right corner indicates the time in minutes from the start of the experiment.

File Name: Supplementary Movie 6
Description: Time lapse movie of the pSTL1-PP7 in a ramp experiment. The leftmost image is a maximum intensity projection of a Z-stack in the green channel allowing to image the PP7-GFP and visualizing the presence of transcription sites as bright foci. The background fluorescence in the image allows to follow NaCl concentration changes in the flow channel. Higher fluorescence is indicative of lower NaCl concentrations. The center-left image is the red channel image and allows to follow the changes in Hog1 nuclear localization. The centerright image is the far-red channel image representing the fluorescence of the Hta2- tdiRFP nuclear tag. The rightmost image is a merged image between the green and red channels (PP7 and Hog1). The number in the lower right corner indicates the time in minutes from the start of the experiment.

File Name: Supplementary Software 1
Description: Script used to extract specific data from PP7 traces. The input is a structure generated by the YeastQuant program (Export File). An example from one data set is provided along the script.
