# Supplementary material for: Single-particle imaging of stress-promoters induction reveals the interplay between MAPK signaling, chromatin and transcription factors
Source: Nat Commun. 2020 Jun 23;11:3171. doi: 10.1038/s41467-020-16943-w (PMC7311541; doi:10.1038/s41467-020-16943-w)
Supplement: Supplementary file 11 — Reporting summary [file 41467_2020_16943_MOESM11_ESM.pdf]

nature research

Corresponding author(s): Serge Pelet

Last updated by author(s): May 8, 2020

Reporting Summary

Nature Research wishes to improve the reproducibility of the work that we publish. This form provides structure for consistency and transparency in reporting. For further information on Nature Research policies, see Authors & Referees and the Editorial Policy Checklist .

Statistics

For all statistical analyses, confirm that the following items are present in the figure legend, table legend, main text, or Methods section.

n/a	Confirmed	
☐	☒	The exact sample size (*n*) for each experimental group/condition, given as a discrete number and unit of measurement
☐	☒	A statement on whether measurements were taken from distinct samples or whether the same sample was measured repeatedly
☐	☒	The statistical test(s) used AND whether they are one- or two-sided<br>*Only common tests should be described solely by name; describe more complex techniques in the Methods section.*
☐	☒	A description of all covariates tested
☐	☒	A description of any assumptions or corrections, such as tests of normality and adjustment for multiple comparisons
☐	☒	A full description of the statistical parameters including central tendency (e.g. means) or other basic estimates (e.g. regression coefficient) AND variation (e.g. standard deviation) or associated estimates of uncertainty (e.g. confidence intervals)
☐	☒	For null hypothesis testing, the test statistic (e.g. *F*, *t*, *r*) with confidence intervals, effect sizes, degrees of freedom and *P* value noted<br>*Give P values as exact values whenever suitable.*
☒	☐	For Bayesian analysis, information on the choice of priors and Markov chain Monte Carlo settings
☒	☐	For hierarchical and complex designs, identification of the appropriate level for tests and full reporting of outcomes
☒	☐	Estimates of effect sizes (e.g. Cohen's *d*, Pearson's *r*), indicating how they were calculated

Our web collection on statistics for biologists contains articles on many of the points above.

Software and code

Policy information about availability of computer code

Data collection	Micro-manager ver 1.4.23
Data analysis	Matlab R2018a, YeastQuant X ver2 (Pelet et al. An integrated image analysis platform to quantify signal transduction in single cells. Integrative biology, 4, 1274–1282 (2012).) Code: [https://github.com/sergepelet/YeastQuantX_V2].

For manuscripts utilizing custom algorithms or software that are central to the research but not yet described in published literature, software must be made available to editors/reviewers. We strongly encourage code deposition in a community repository (e.g. GitHub). See the Nature Research guidelines for submitting code & software for further information.

Data

Policy information about availability of data

All manuscripts must include a data availability statement. This statement should provide the following information, where applicable:

- Accession codes, unique identifiers, or web links for publicly available datasets
- A list of figures that have associated raw data
- A description of any restrictions on data availability

Source data for Figures 1d, 2a, 2f,2g, 3a, 4b, 4c, and 6c and Supplementary Figures 1, 4, 5, 6, 8, 12, 13. The datasets generated and analyzed during the current study are available from the corresponding author on reasonable request.

Field-specific reporting

Please select the one below that is the best fit for your research. If you are not sure, read the appropriate sections before making your selection.

Life sciences study design

All studies must disclose on these points even when the disclosure is negative.

Sample size	At least 100 single cell measurements per replicate but often as many as 200 to 400. This number is limited by the acquisition settings use throughout the study where an optimum has to be found between the number of field of view imaged (=> more cells measured) and the speed of the data acquisition (temporal resolution of the data set). With hundred single cell measurements, we start to be able to make statistically robust assessments.
Data exclusions	Replicate were discarded if light source instability were encountered. Some data sets were also excluded based on cell size adaptation dynamics. The addition of the stress medium in each well is done manually and can sometimes lead to some unusual adaptation behavior. Based on a large number of experiments performed with WT cell, we know what is the expected cell adaptation behaviors and experiments deviating too much from this mean response were discarded.
Replication	All experiments were performed in fully independent duplicates for non-induced samples and triplicate (at least) for induced samples. In the triplicate datasets some of the experiments had to be discarded due to cell adaptation behavior problem. Otherwise all replicate gave similar results.
Randomization	Experiments were always performed with different starting cultures and often imaged on different days.
Blinding	The experimentalist knew the strain and the stress level applied. However, all dataset were analyzed in an automated fashion without user intervention.

Reporting for specific materials, systems and methods

We require information from authors about some types of materials, experimental systems and methods used in many studies. Here, indicate whether each material, system or method listed is relevant to your study. If you are not sure if a list item applies to your research, read the appropriate section before selecting a response.

Materials & experimental systems

n/a	Involved in the study	
☒	☐	Antibodies
☒	☐	Eukaryotic cell lines
☒	☐	Palaeontology
☒	☐	Animals and other organisms
☒	☐	Human research participants
☒	☐	Clinical data

Methods

n/a	Involved in the study	
☒	☐	ChIP-seq
☒	☐	Flow cytometry
☒	☐	MRI-based neuroimaging
