# Supplementary material for: Single-particle imaging of stress-promoters induction reveals the interplay between MAPK signaling, chromatin and transcription factors
Source: Nat Commun. 2020 Jun 23;11:3171. doi: 10.1038/s41467-020-16943-w (PMC7311541; doi:10.1038/s41467-020-16943-w)
Supplement: Supplementary file 12 — Source Data [file 41467_2020_16943_MOESM12_ESM.zip › SourceData/ReadMe.pdf]

Source data for Wosika et al. Nat Com 2020

The data provided here should be sufficient to generate the graphs found in the publication. A more complete data set and the raw data can be obtained by contacting the authors of the publication. Below is a short explanation of the data provided. Also refer to the Supplementary Tables for a list of the provided data sets.
All files are named according to the following notations:
- Figure_Strain_Perturbation_Replicate_Channel_Measurement.csv

or simply:
- Figure_Strain_Perturbation_Replicate_Measurement.csv

Measurement Name	Size	Explanation
Time	Nb of time point x 1	vector containing the reference time of the measurements in minutes
CellLabel	1 x Nb of cells	Tracking label for each single cell.
HiPix	Nb of time point x Nb of cells	Matrix containing the difference between the 20 Highest Pixels in the ExpNucl object and the average intensity of the ExpNucl object
Connected HiPix	Nb of time point x Nb of cells	Matrix containing the difference between the identified ConnectedHiPix in the ExpNucl object and the average intensity of the ExpNucl object. Filtering of the object for isolated ConnectedHiPix was performed
Area	Nb of time point x Nb of cells	Size in pixels of the Cell Object
NuclCytoDiff	Nb of time point x Nb of cells	Difference between the average intensity in the nucleus and in the cytoplasm objects
NuclCytoRatio	Nb of time point x Nb of cells	Ratio between the average intensity in the nucleus and in the cytoplasm objects
Media	Nb of time point x Nb of cells	Average intensity in an object surrounding the cell object. Allows to monitor the background fluorescence of the medium
